# Cultivated meat microbiological safety considerations and practices

**DOI:** 10.1111/1541-4337.70077

**Published:** 2024-12-28

**Authors:** Dean Joel Powell, Dan Li, Ben Smith, Wei Ning Chen

**Affiliations:** ^1^ The Good Food Institute Asia Pacific (GFI APAC) Singapore Singapore; ^2^ School of Chemistry, Chemical Engineering and Biotechnology Nanyang Technological University Singapore Singapore; ^3^ Department of Food Science and Technology National University of Singapore Singapore Singapore; ^4^ Bezos Center for Sustainable Protein National University of Singapore Singapore Singapore; ^5^ Monell Chemical Senses Center Philadelphia Pennsylvania USA; ^6^ Future Ready Food Safety Hub (FRESH) Nanyang Technological University Singapore Singapore

**Keywords:** alternative proteins, cultivated meat, cultured meat, microbial contamination, microbiological testing

## Abstract

Cultivated meat, produced using cell culture technology, is an alternative to conventional meat production that avoids the risks from enteric pathogens associated with animal slaughter and processing. Cultivated meat therefore has significant theoretical microbiological safety advantages, though limited information is available to validate this. This review discusses sources and vectors of microbial contamination throughout cultivated meat production, introduces industry survey data to evaluate current industry practices for monitoring and mitigating these hazards, and highlights future research needs. Industry survey respondents reported an average microbiological contamination batch failure rate of 11.2%. The most common vectors were related to personnel, equipment, and the production environment, while the most commonly reported type of microbiological contaminant was bacteria. These will likely remain prominent vectors and source organisms in commercial‐scale production but can be addressed by a modified combination of existing commercial food and biopharmaceutical production safety systems such as Hazard Analysis and Critical Control Points (HACCP), Good Manufacturing Practices (GMP), and Good Cell Culture Practice (GCCP). As the sector matures and embeds these and other safety management systems, microbiological contamination issues should be surmountable. Data are also included to investigate whether the limited microbiome of cultivated products poses a novel food safety risk. However, further studies are needed to assess the growth potential of microorganisms in different cultivated meat products, taking into account factors such as their composition, pH, water activity, and background microflora.

## 1 INTRODUCTION

Cultivated meat, produced using cell culture technology, comprises some or all of the critical cell types of meat with the potential to replicate the sensory and nutritional profile of conventionally produced meat, seafood, or organ meat. Cultivated meat builds upon decades of accumulated knowledge in cell culture, stem cell biology, tissue engineering, fermentation, and bioprocess engineering used by related industries to produce safe and trusted drugs and medicines used throughout the world (Specht et al., 2018). Cultivated meat production offers potential environmental advantages compared to conventional meat production such as significantly lower land use, air pollution, and greenhouse gas emissions—especially when sustainable energy is used (Sinke et al., 2021, 2023; Tuomisto, 2019; Tuomisto et al., 2022).

**FIGURE 1:**
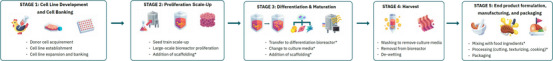
Five‐stage generalized bioprocess of cultivated meat production. Asterisk indicates that inputs may vary by individual bioprocess or not be used at all.

As reviewed by Sant'Ana et al. (2023), most cultivated meat manufacturers follow roughly the same bioprocess, which is grouped into five main stages for this review (Figure 1). In brief, cells are acquired from a healthy animal and undergo a process of isolation and selection to develop a high‐performing cell line (Stage 1). The cell lines are grown in a series of flasks and/or bioreactors of increasing size called the seed train, until they reach sufficient numbers to seed into a large‐scale proliferation bioreactor while being fed a cell culture medium made primarily of basic nutrients such as amino acids, glucose, salts, and vitamins (Stage 2). Changes in the medium composition and/or bioreactor environment trigger the transformation of these cells into mature cells such as fat and skeletal muscle (Stage 3). The mature cells are then harvested (Stage 4) and formulated, processed, and packaged (Stage 5) into final products (Sinke et al., 2023).

The production of cultivated meat does not require animal slaughter, except potentially for initial cell isolation at Stage 1, which is separate from the food production process during Stages 2–5. Therefore, from a food safety perspective, contamination from animal‐derived pathogens such as enteric zoonotic bacteria (e.g., pathogenic *Escherichia coli*, *Salmonella*, *Campylobacter*) that lead to common foodborne illnesses is eliminated de facto. This can be compared to conventional meat production where data from the European Food Safety Authority (EFSA) indicate that conventional meat, seafood, and meat‐based products were responsible for 24.4% of foodborne disease cases (that were identified with strong evidence) in reporting EU countries in 2017 (EFSA and ECDC, 2018). Grown in a contained, controllable, and nominally sterile process with minimal to no use of animal‐derived ingredients (outside of cells and potentially some culture media components), cultivated meat can, from a technical feasibility perspective, replicate sterile cell culture similar to that employed by the pharmaceutical sector to virtually eliminate contamination with disease‐causing pathogens (Rubio et al., 2020). In this vein, most companies in the cultivated meat industry are developing antibiotic‐free production processes, according to a cultivated meat bioprocess survey by Harsini and Swartz (2024), while the initial products already approved for sale have been produced without antibiotics, as indicated by the cell culture consultation dossiers submitted to the US Food and Drug Administration (FDA) by GOOD Meat Inc. and UPSIDE Foods, as well as the documentation submitted by Vow Foods to Food Standards Australia New Zealand (FSANZ).

Food safety competent authorities are challenged to follow these advances and create the basis for regulatory authorization with the necessary responsiveness to cover the innovative and varied cultivated meat production methods being developed while ensuring that the produced food is safe for the consumer. Thus, it is important to understand the potential microbiological contamination risks and vectors for cultivated meat production to appreciate what production strategies may be appropriate. This review aims to outline relevant microbiological contamination risks for cultivated meat and provide information on current industry practices and thinking around microbiological contamination prevention and testing based on the available literature, other relevant industries, and unpublished industry‐sourced data presented below.

## 2 REVIEW METHODOLOGY

As cultivated meat is an emerging industry, relatively few original research papers have addressed the technology's microbiological risk and testing considerations. Relevant literature and general principles of microbiological risk assessment can be derived from conventional meat production and related adjacent fields, such as mammalian cell culture for biopharmaceutical applications or industrial fermentation in the food industry.

**FIGURE 2:**
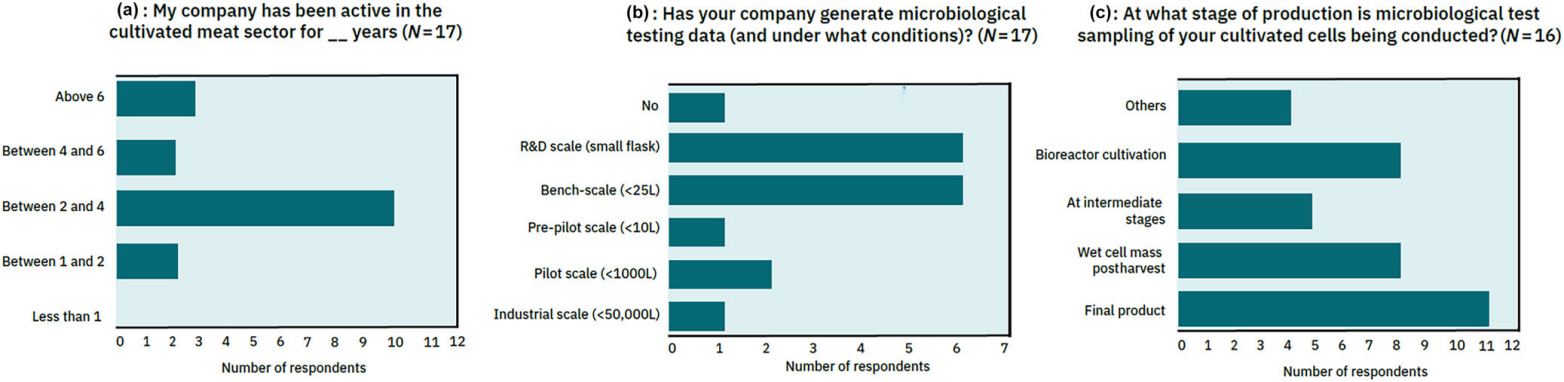
Cultivated meat industry survey company profile. (a) Cultivated meat company duration of activity in the sector. Number of respondents = 17. (b) Cultivated meat company microbiological testing practices by largest relevant scale of production. Number of respondents = 17. (c) Cultivated meat company microbiological testing practices by stage of production. Stages indicated as “Other” were during cell line generation (x2), in master and working cell banks (x1), and during R&D cultivation (x1). Number of respondents = 16, and respondents were allowed to select as many options as relevant.

**FIGURE 3:**
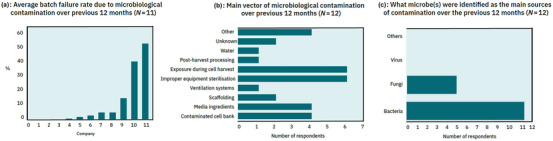
Cultivated meat industry survey microbiological contamination trends. (a) Cultivated meat company batch failure rate over the previous 12 months as a result of microbiological contamination. Companies 1, 2, 3, and 7 indicated microbiological testing had only been conducted at R&D scale (small flask, grams of biomass per lot). For responses provided by respondents as “<x%”, “x%” was recorded. Number of respondents = 11. (b) Cultivated meat company identification of the main process or input vector of microbial contamination for their cultivated meat production over the previous 12 months. Respondents were allowed to select up to three options where appropriate, based on production in their largest scale system. “Other” sources identified were improper handling (x2), improper bioreactor design, and “source material.” Number of respondents = 12. (c) Cultivated meat company identification of the main microbe source of contamination for their cultivated meat production. Respondents were allowed to select multiple options where appropriate. Number of respondents = 12.

**FIGURE 4:**
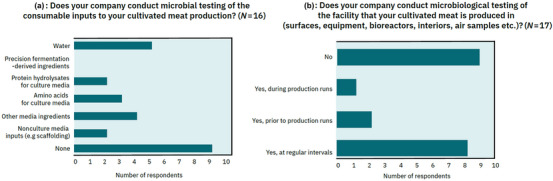
Cultivated meat industry survey microbiological testing other than end‐products. (a) Cultivated meat company microbiological testing of consumable inputs by category. Respondents were allowed to select multiple options where appropriate. Number of respondents = 16. (b) Cultivated meat company microbiological testing of the facility in which they conduct cultivated meat production. Respondents were allowed to select multiple options where appropriate. Number of respondents = 17.

**FIGURE 5:**
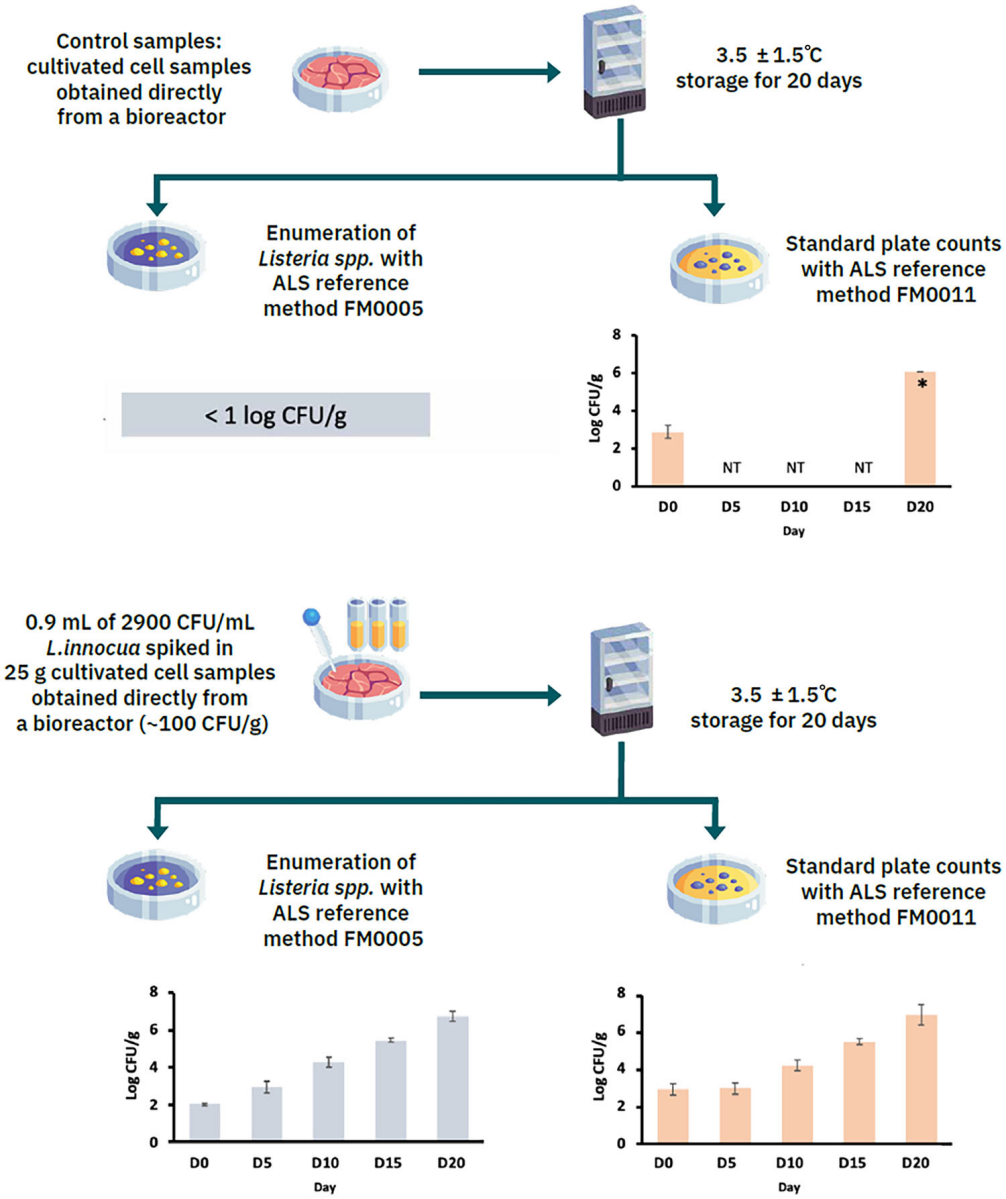
Experimental setup and results of the challenge study of *Listeria innocua* in cultivated meat products provided by an anonymized cultivated meat company. Each data point represents the average of three independent batches of samples ± standard deviations (SD). NT, not tested. Asterisk indicates results from Batch 2, whereas Batches 1 and 3 generated results below 1 log CFU/g.

To supplement this literature and documentation of principles from other industries, this review incorporates information obtained through a targeted online survey of cultivated meat manufacturers regarding their microbiological testing activities (with 17 unique cultivated meat‐producing company respondents) conducted between May and July 2023 (Figures 2, 3, 4). As of the end of 2023, there were 174 publicly announced cultivated meat companies including both suppliers as well as cultivated meat production companies (Battle et al., 2024), indicating our survey reached 10% of the global sector. Respondent companies ranged from being in operation from 1–2 years through to >6 years (Figure 2a). Survey results included information regarding the scale and point of production where microbial testing of cultivated cells occurs (Figure 2b,c), batch failures due to microbial contamination over the previous 12 months (Figure 3), and microbial testing of consumable inputs and the production environment (Figure 4). Multiple‐option selection was allowed for many survey questions, and the respondents could skip questions that they felt were commercially sensitive. All information was shared on the requirement that it only be included in this review in a de‐identified and aggregated format. In addition, one cultivated meat company also shared their unpublished microbiological challenge study dataset of *Listeria innocua* in cultivated poultry cell mass at the postharvest (storage) stage (Figure 5), which was conducted by a third‐party accredited food microbiological laboratory (company and product name not revealed at the request of the supplying company).

## 3 PUTATIVE MICROBIOLOGICAL SAFETY ADVANTAGES AND RISKS OF CULTIVATED MEAT PRODUCTION

Broadly, the main contribution of microbiological contamination in conventional meat production is between the farm and the chilling of the carcass at the slaughterhouse, with contaminated feces or hide at the farm and contaminations associated with carcass dehiding, evisceration, or splitting at the slaughterhouse being the primary contributing factors (Tesson et al., 2020). Muscle tissue in healthy live animals is sterile but is inevitably exposed to microbes during slaughter and processing, forming a microbiome on meat products (Lawrie & Ledward, 2014). In comparison, cultivated meat production from Stage 1 through Stage 3 (Figure 1) has the potential to be produced under well‐controlled conditions of sanitation in closed‐loop stainless steel bioreactor systems, uses cells subjected to rigorous screening procedures to exclude microbial contamination, and is not exposed to any animal‐based contamination during processing at Stage 4 or 5 (FAO, 2022; Ong et al., 2021). A survey of 24 cultivated meat companies by Harsini and Swartz (2024) found that 79% expect the microbial contamination risk to be lower for cultivated meat than for conventional meat products. Harsini and Swartz (2024) also asked cultivated meat companies with operational pilot/production facilities (*n* = 6) about their all‐cause batch failure rate (including contamination, equipment failure, etc.). Of the six companies with operational pilot/production facilities, three reported batch failure rates between 2% and 5%, two reported rates between 5% and 10%, and one reported a rate exceeding 10%.

To specifically compare the theoretical microbial contamination prevention advantages of cultivated meat production to current industry experience, our targeted industry survey asked companies to disclose their average cultivation batch failure rate due to microbiological contamination exclusively, for all production runs in the previous 12 months. Of 11 responses, the batch contamination rate over the previous 12 months varied substantially from 0% up to 52%, with an average of 11.2% (Figure 3a). When the responses of companies that had only conducted cultivation at the R&D scale are ignored—which was characterized in the survey question as cells cultivated in a small flask with grams of biomass per lot—the average batch contamination rate for the remaining six respondents was much higher at 19.5%. This likely reflects the increased complexity of contamination control in larger facilities and equipment that is more reflective of a commercial facility.

Given that these potential microbiological safety advantages compared to conventional meat are yet to be verified with the operation of large‐scale cultivated meat production facilities, it can be instructive to look at relevant adjacent industries. Specifically, many similarities exist between the bioprocess employed by cultivated meat manufacturers and the biopharmaceutical industry's production of monoclonal antibodies using mammalian cell culture. Methods routinely employed for the biopharmaceutical manufacturing sector were estimated to have a batch failure once every 58 weeks on average, of which contamination‐associated failures contributed about 3.2% at commercial facilities in 2022 (Ranck, 2022). This total batch failure rate has seen a steady improvement from one failure every 40 weeks per facility on average in 2008 (Ranck, 2022), highlighting the importance of long‐term operation experience for process optimization.

Applying this lens to cultivated meat, our industry survey results as well as those of Harsini and Swartz (2024) clearly indicate that the cultivated meat industry is yet to realize the microbiological safety potential that the technology is, in theory, capable of through Stages 1–4 of production based on the industrial‐scale mammalian cell culture example of the biopharmaceutical industry. Indeed, it is likely that the full potential of the microbiological safety advantages of cultivated meat will take years of commercial operational experience to maximize. Once standardized processes, input sourcing, training, and monitoring are well established, alongside the potential use of predictive microbiological models, biofilm prevention protocols, and advanced pathogen detection systems, microbial contamination rates are expected to decrease significantly. Therefore, techniques to maintain the sterility of cultures may be important considerations in the short to medium term to improve the consistency of operations (McNamara & Bomkamp, 2022). Additionally, as the production of cultivated meat scales up, it is unlikely that comprehensively replicating the pharmaceutical industry's sterility practices will be an economically feasible approach for food production due to differences in the viable cost of production. For context, a recent estimate of the cost of production that modern monoclonal antibody production could achieve was $50,000/kg (Kelley, 2024), whereas cultivated meat production will ideally aim to compete with conventional animal product pricing as highlighted by Pasitka et al. (2024), who modeled the feasible production of a 50% cultivated chicken product for $13.7/kg. Alternatively, some companies may look toward the use of food‐grade antimicrobial agents (Gyawali & Ibrahim, 2014) or antimicrobial small molecules (Ghosh et al., 2014) that could be employed alongside good hygiene practices to mitigate contamination risk. So far, some research on this topic has been initiated, such as the Bioengineering Tools for Next‐Generation Cellular Agriculture (CELLAG) project jointly funded by Singapore and Israel to develop sensors for microbial contamination detection and novel antimicrobial compounds specifically for cultivated meat applications (Abdullah & Klein, 2023). However, additional work on this topic could address a wider range of potential technologies to apply for this purpose.

In biopharmaceutical manufacturing, aseptic conditions are maintained through the enveloping design of the bioreactor, operation under positive pressure, strict adherence to aseptic technique during manipulations, and the use of sensors such as those for pH or specific metabolites (Ali et al., 2020; Djisalov et al., 2021). The FDA cell culture consultation dossier from GOOD Meat indicates that the company is following many of these aseptic manufacturing processes. For example, GOOD Meat states in their dossier that the processes of cell expansion, centrifugation, washing, and packaging take place in a clean room with controlled air quality, high‐efficiency particulate air (HEPA) filters, and differential air pressure as designed for the biopharmaceutical industry (Licari, 2022).

Whether such processes remain the norm is to be seen, especially considering the associated cost. Classified clean rooms may not be necessary for some, or all, stages of production with improvements to manufacturing equipment design, such as the use of closed systems rather than controlled environments (Bevan et al., 2021). It has similarly been suggested that other standard sterility practices in biopharmaceutical manufacturing, such as strict employee gowning requirements, may be redundant and avoided in establishing safe and cost‐effective cultivated meat production protocols (Croughan et al., 2014). In contrast to GOOD Meat, Dutch cultivated meat company Mosa Meat stated in a blog on November 10, 2021 that “The meat harvesting process … would likely be in an [International Standards Organization] ISO Class 8 area” (Mosa Meat, 2021). ISO 8 is the least stringent clean room classification that requires HEPA‐filtered air. Similarly, UPSIDE Foods stated in its FDA cell culture consultation dossier that “all operations will be performed in clean equipment under temperature‐controlled conditions,” suggesting that they do not use a classified clean room environment for this stage of production (Schulze, 2021).

Application of Good Cell Culture Practice (GCCP) as outlined by Coecke et al. (2005), and updated by Pamies et al. (2022), has provided a starting point for safe production in lieu of ISO standards for cultivated meat production. It has been suggested that the complete set of standards outlined by GCCP is likely prohibitive for food production but that the development of “food‐grade” GCCP based on these existing guidelines could be undertaken (Ong et al., 2021) to leverage relevant practices developed for the biopharmaceutical sector.

## 4 MICROBIAL CONTAMINATION VECTORS IN THE PRODUCTION PROCESS

Identification and collation of potential points of entry for microbial contamination in the cell cultivation process may vary based on a specific company's bioprocess and can be mapped through the Hazard Analysis and Critical Control Points (HACCP) identification methodology (Hazard analysis and critical control point principles and application guidelines. Adopted August 14, 1997. National Advisory Committee on Microbiological Criteria for Foods, 1998). As an example, Sant'Ana et al. (2023) published a food safety plan and hazard analysis for a theoretically cultivated burger production process broken down by HACCP methodology into each component process step and input used. However, there are many mandatory process steps, types of equipment, and inputs for cultivated meat production that could be assessed on an industry‐wide basis for relevant microbiological contamination vectors and controls and published as standards or guidelines.

Respondents to our survey identified improper equipment sterilization (*n* = 6) and exposure during cell harvest (*n* = 6) as the most common vectors of microbiological contamination in their operations (Figure 3b). Similarly, Harsini and Swartz (2024) found that of 22 cultivated meat companies surveyed, a majority identified process contamination—such as equipment issues, sampling, adding culture components, or harvesting—as the main vector of microbial contamination in cultivated meat production (*n* = 13), followed by final product contamination (*n* = 7). Together, these results suggest that personnel (such as staff introducing microbes through poor personal hygiene or infection), equipment (such as improper cleaning or sterilization of food contact surfaces), and the environment are the main contamination vectors that cultivated meat companies currently need to address by improving their processes and training. This conclusion is supported by FSANZ documentation for the regulatory review of Australian cultivated meat company Vow, which reportedly identified the main potential vector for microbial contamination as the harvesting and postharvest handling of the cells—mainly from food contact surfaces, equipment, and personnel (FSANZ, 2023).

This will likely result in a similar trend to plant‐based meat analog production where microbial hazards from livestock are avoided but others can be introduced through personnel‐based vectors, or improper hygienic design and inadequate cleaning of equipment, notably during the processing stage where *Staphylococcus aureus* or viruses can be introduced through food handling, or *Listeria monocytogenes* may be introduced from the production environment (Banach et al., 2023).

A contributing factor to these contamination incidents may be that only a minority of respondent companies in our survey reported having a HACCP plan in place at the time of our survey (*n* = 7), while a similar amount had one under development (*n* = 6) and a small minority had not initiated the process of developing a HACCP plan (*n* = 3).

Overall, the responses to the main sources of microbiological contamination in our survey (Figure 3b) can be broken down into three major categories of contamination vectors—cell line based (4); media and consumable inputs (7); and personnel, equipment, and environment (17). The following sections will therefore discuss these three major categories of contamination vectors and how they relate to the five stages of cultivated meat production as per Figure 1.

### 4.1 Cell line vectors

In Stage 1 of production, an existing cell line is acquired from an outside source (e.g., a cell line repository), or a tissue biopsy or other biological sample (e.g., egg, blood, or feather) is taken from a living or recently slaughtered animal. The sample is taken to a laboratory, where the tissue biopsy is processed to isolate a specific cell population; the cells are then expanded using two‐ or three‐dimensional culture methods, selected or engineered for improved production characteristics, characterized, and finally preserved in a cryopreservation medium for cell banking to subsequently be used in production (Ong et al., 2021; Zehorai et al., 2023).

Cell line sampling, selection, and banking are the first potential sources of microbial contamination in cultivated meat production. Zoonotic infectious diseases and foodborne pathogens can be transmitted from the source animal or their feces through the biopsy to the resulting cells selected for cultivation (FAO & WHO, 2023). However, the risk of this is noted to be considerably lower compared to conventional livestock breeding (Treich, 2021). This is supported by our survey results where cell lines were the least reported category of microbial contamination during cultivated meat production (*n* = 4; Figure 3b). Another potential vector, specific to viral contamination, is the use of viral construct‐mediated genetic modification to improve a cell line's characteristics, as discussed in detail in Section 6.3.

The risk of contamination with adventitious agents can be minimized by preventative controls, such as obtaining cells that originate from donor animals in good health and, when possible (depending on species), raising them in closed flocks, herds, or colonies that are confirmed to be specific‐pathogen‐free. In some cases, an inspection by a veterinarian as a pre‐ and/or postmortem inspection (if the cells are acquired post‐slaughter) can assist in making determinations of an animal's health (FAO & WHO, 2023; Melzener et al., 2021), although this is not routine for some animal species (e.g., those used for seafood). Selection and testing criteria related to cell sourcing have been considered previously for relevant industries, such as viral vaccine manufacturers, and can be leveraged for cultivated meat production (FDA, 2010).

### 4.2 Cell culture media and other consumable input vectors

Cell culture media or its component ingredients are another potential microbial contamination vector relevant to Stages 1–3 of production. Cell culture media are a complex mixture of salts, sugars, vitamins, amino acids, organic acids, growth factors, and hormones (O'Neill et al., 2021), which can be supplied to companies as powdered raw ingredients that can be reconstituted in filtered water on site, or it may be provided as a complete liquid formulation prepared by a third party supplier. The use of animal‐derived serum or media components can also increase the risk of contamination by viruses and infectious prions (Hadi & Brightwell, 2021; Ong et al., 2021). Although most cultivated meat companies have signaled that they have removed, or will in the future eliminate, animal‐derived media ingredients from their media formulations, animal serum has been included in the cultivated chicken media formulations of UPSIDE Foods and GOOD Meat that received regulatory approvals. Additionally, in 2023, the company Omeat announced its intent to supply ethical and sustainably sourced cow serum as an ingredient for the cultivated meat sector, rather than remove it completely (Naureen, 2023).

Sterilization of the complete media prior to its addition to any cell culture or bioreactor can be achieved using one of a variety of methods that have been applied in related industries or the food industry. These include filtration, irradiation, pulsed electric fields, and/or high‐temperature, short‐time (HTST) pasteurization. Methods vary in their ability to capture or eliminate certain adventitious agents and in some cases may be inappropriate for use due to heat‐labile ingredients in the cell culture media formulation. These sterilization methods reduce the risk of contamination from adventitious agents, which sometimes can originate from nonanimal media components (Barone et al., 2020).

In general, any component of the cell culture medium in Stages 1–4 of production has the potential to expose cells to microbiological contamination (Ong et al., 2021). This can be extended to scaffolding materials, processing aids, as well as food contact materials such as single‐use bioreactors and bioreactor bags. Suppliers can operate their facilities under relevant food or pharmaceutical Good Manufacturing Practices (GMP) guidelines to demonstrate to cultivated meat producers that controls are in place to prevent unintentional upstream contamination with microbiological hazards that could accompany their product during the cell cultivation process. Highlighting the importance of supplier practices, of 16 industry survey respondents, 56% did not conduct or contract microbiological testing of their consumable inputs (Figure 4a), instead relying on supplier practices. Additionally, of the 12 industry survey respondents that reported their main vectors of microbiological contamination over the previous 12 months, 23% of the 31 nominated primary vectors were related to media and consumable inputs (specifically media ingredients, scaffolding, and water; Figure 3b).

An additional type of input for most, if not all, cultivated meat companies in Stage 5 of production will be traditional food ingredients, such as plant proteins, binders, and flavors, which are an important vector to incorporate into a cultivated meat hazard analysis. For example, despite *Bacillus cereus* being a pathogen that can be associated with conventionally derived meat (Tewari et al., 2015), Sant'Ana et al. (2023) considered it as a potential biological hazard in their cultivated beef burger HACCP study primarily due to some plant‐based ingredients being used in the process and the likelihood of biofilm formation (Akamatsu et al., 2019; Ellouze et al., 2021; Lin et al., 2022; Majed et al., 2016).

### 4.3 Personnel, equipment, and environmental vectors

The third general category of microbial contamination vectors includes personnel, bioaerosols that may be present in ambient air (airborne microorganisms, dust, pollen, and mold spores), biofilms, equipment, and utensils (Shilenge et al., 2017). Exposure to these vectors can occur during all stages of production, for example, during the handling of tissue samples, transferring of cells, thawing of cells in water or bead baths, cell storage, and cell culture that occurs using plastic dishware in Stage 1; preparation, operation, and cleaning of bioreactors and other equipment during Stages 1–3; harvesting, washing, and de‐wetting of cells during Stage 4; and handling and processing of cells into final products during Stage 5 (FAO & WHO, 2023; Ong et al., 2021).

Although the bioreactor environment would be closed during Stages 2 and 3 of production, there is always a risk of contamination during operation due to process failures such as improper equipment sterilization or environmental control failures. This is especially so in less automated production facilities or those that employ a batch or semicontinuous bioprocess where bioreactors have a higher degree of exposure to the external environment and present an increased opportunity for contamination from personnel‐based vectors, improper hygienic design, or inadequate cleaning of equipment.

The presence of potential microbial contaminants can be monitored throughout the Stage 2 and 3 bioprocess based on potential indicators of cytopathic effects on the cell substrate. The effects may be recognizable by monitoring on‐line, at‐line, or off‐line optical density measurements, dissolved oxygen levels, metabolite levels, pH indicators, morphological assessment, or other desired parameters over time (Fung Shek & Betenbaugh, 2021). It should be noted that at‐line or off‐line sampling is often associated with a higher risk of process contamination; therefore, online sensors are preferable (Beutel & Henkel, 2011; Claßen et al., 2017; Reyes et al., 2022). Harsini and Swartz's (2024) industry survey found that 17 out of 23 respondent companies were using a combination of online and offline bioprocess monitoring, with only four exclusively using online monitoring, indicating that this is an area where microbial contamination risk could be lowered.

Routine contamination testing for specific bacteria, fungal, protozoa, and parasite strains should be redundant at Stages 2 and 3 if process monitoring is conducted appropriately, and contamination testing for relevant microbes is conducted at Stage 1. However, some microorganisms grow slowly and have long incubation periods, and the aforementioned bioprocess monitoring methods may be insufficient for their detection. *Mycoplasma* contamination is an example that can present a food safety risk and generally requires different testing compared to other bacteria as they may be largely undetectable through typical process monitoring (Armstrong et al., 2010; Chandler et al., 2011). See Section 6.1.4 for additional discussion of *Mycoplasma* contamination and testing considerations.

Separate from microbial contamination testing of the cell culture, routine testing of the production environment and surfaces is another important facet of monitoring. Of 17 industry survey respondents, only 48% reported that they conduct microbial testing of their production facility, such as surfaces, equipment, bioreactor interiors, air samples, and so forth (Figure 4b). This relatively low rate of testing could have contributed to the high proportion of contamination events due to personnel‐, equipment‐, and production environment‐related vectors, particularly improper equipment sterilization (Figure 3b), as such testing is recommended under relevant food or pharmaceutical GMP.

Harvesting, washing, and de‐wetting of the cell mass during Stage 4 of production present potential personnel, equipment, and environmental vectors of contamination for pathogens such as *L. monocytogenes* and *Salmonella* that can be compared to those conventional meat is exposed to during carcass evisceration and splitting, excluding crucial animal‐derived vectors such as dirt‐contaminated skin, hide or feathers; gastrointestinal tract contents; and feces (Tesson et al., 2020). Both the cultivated cell mass and conventional meat tissue are nominally sterile until this stage when the bioreactor seal and animal carcass integrity are intentionally breached, respectively (Lawrie & Ledward, 2014). Despite the removal of animal‐derived vectors, 50% of survey respondents indicated exposure during cell harvest was one of their main vectors of microbiological contamination over the previous 12 months (Figure 3b), highlighting that appropriate microbiological contamination management and testing at Stage 4 are critical considerations.

Many of the microbiological contamination vectors in Stage 5 (mixing of the cultivated cells with other food ingredients, molding and forming the cells into specific product formats, and packaging) will be similar to those observed in conventional meat production during the secondary processing of meat into specific cuts, ground meat, formed products, or various ready‐to‐eat (RTE) products. The cell mass will experience many of the same types of exposure to personnel and the production environment, as well as the same pathogens such as *L. monocytogenes* and *Salmonella*, as outlined by Sant'Ana et al. (2023). Some cultivated meat production may even use the same equipment and facilities as conventional meat production, as occurred when GOOD Meat used the Food Innovation and Resource Centre's food pilot production facility in Singapore for its first commercialized cultivated chicken product (FAIRR, 2021). These are not novel challenges, and to manage these contamination risks, the sector can borrow and adapt GMP and HACCP knowledge and practices from conventional meat and other processed food production processes, in order to reduce contamination incidence during Stage 5 of production.

## 5 CULTIVATED MEAT MICROBIOME AND MICROBIOLOGICAL ACTIVITY POSTPRODUCTION

The US cultivated meat companies UPSIDE Foods and GOOD Meat demonstrated commercial sterility of their cultivated meat, reporting no detectable microorganisms using aerobic plate counts, an absence of coliforms, *E. Coli*, mold, and yeast on a per gram basis, and an absence of *Campylobacter* in a 25‐g sample as outlined in their cell culture consultation premarket safety notices to the FDA (Licari, 2022; Schulze, 2021). These test results are almost certainly generated from samples obtained at Stage 4 of production immediately postharvest, as cultivated meat will be exposed to many of the same vectors of contamination as conventional meat at Stage 5 of production, leading to the formation of a microbiome on the final cultivated food product.

However, it has been suggested that sterile cultivated meat products could be more susceptible to newly colonizing pathogens that may reach the finished product in Stages 4 and 5 of production through error or chance (Lawton, 2020), without the requisite exposure to nonpathogenic microbes from slaughtered animals that form the standard microbiome for conventional meat during processing. For example, *S. aureus* is normally outcompeted by the microbiome present in raw food products and is therefore normally only involved in food poisoning incidents through contamination of foods that have had their microbiome previously minimized through cooking or processing (Shawish & Al‐Humam, 2016). To better understand this risk, shelf life studies or microbiological challenge studies will be instructive to determine how exposure to pathogens from open air during production, shipping, storage on shelves, refrigeration, and so forth may affect the products and their shelf life (Ong et al., 2021). UPSIDE Foods outlined their approach of judging “acceptable” sensory quality as well as microbial limitations for establishing their product shelf life guidelines in their FDA cell culture consultation dossier (Schulze, 2021) but did not divulge their experimental methodology or data.

Results from one such unpublished study, provided by a cultivated meat company on condition of anonymity, are thus included in this paper (Figure 5). The study investigated *Listeria* spp., which are ubiquitous in the natural environment and of which *L. monocytogenes* is an important hazard for processed meat and poultry products, which have a well‐established association with the risk of foodborne listeriosis (Ross et al., 2009; WHO, 2004). The cultivated uncooked poultry cell samples were obtained directly from a bioreactor from three separate production batches with an average pH of 6.30 ± 0.27. They were inoculated with *L. innocua* (strain (LI01) ATCC 33090/NCTC 11288), a nonpathogenic surrogate with similar characteristics to *L. monocytogenes*, at approximately 100 CFU/g and stored at 3.5 ± 1.5°C for up to 20 days. The samples were tested for *Listeria* spp. and standard plate count (SPC).

*Listeria innocua* showed continuous growth throughout the 20‐day storage period, increasing from an initial concentration of 2.0 ± 0.1 to 6.8 ± 0.3 log CFU/g (*p* < .05; Figure 5). The maximum growth rate was calculated as 0.253 ± 0.006 log/day with the use of the DMFit tool at ComBase (Baranyi and Roberts Model, *R* ^2^ = .999, SE = 0.060). Previous studies that have investigated *L. monocytogenes* growth in raw or partially cooked poultry under similar temperature and atmospheric storage conditions (Lianou et al., 2021; Mano et al., 1995; Shineman & Harrison, 1994; Wimpfheimer et al., 1990) have demonstrated both higher and lower growth dynamics compared to the cultivated poultry product, suggesting that its reduced microflora did not support *Listeria* spp. growth dynamics that could not otherwise occur in conventional poultry.

However, these results and previous studies do highlight the established and complex interaction of factors that are known to influence *Listeria* spp. growth, including not only temperature and pH, but also product composition, microstructure, water activity, and fat content (Dykes, 2003; Hwang & Tamplin, 2007; Verheyen et al., 2019), which were not consistently reported or controlled in the above *L. monocytogenes* studies.

The challenge study also included control samples without *L. innocua* inoculation to assess SPC in the cultivated poultry cell batch and to monitor any potential *Listeria* spp. contamination that may have occurred prior to the inoculation step. Initial analyses of the SPC in all three batches of cultivated cells revealed an average of 2.9 ± 0.3 log CFU/g (Figure 5). However, *Listeria* spp. were not detected in any of the control samples at the end of the study, confirming the absence of previous contamination and validating the results of the challenge study.

During the 20‐day cold storage period, the control samples showed varying trends in SPC. While Batch 1 and Batch 3 showed a decrease in bacteria to undetectable levels (less than 1 log CFU/g), Batch 2 showed a significant increase to 6.1 log CFU/g by Day 20. This increase in SPC in Batch 2, although not attributed to *Listeria* spp., approaches or exceeds the limits of acceptability established by various food safety agencies for meat products (Kim et al., 2018). For instance, according to FSANZ, the normal background microflora for raw commodities at the end of shelf life such as meat and fish can range from an SPC of 6 to 7 log CFU/g (FSANZ, 2022). Similarly, the Singapore Food Agency (SFA) allows a similar range for chilled or frozen blended meat (Singapore Food Agency, n.d.). This observation in Batch 2 requires further investigation to identify the specific microorganisms responsible for this increase as this would dictate their potential spoilage mechanisms (Snyder et al., 2024).

Comparing the initial SPC data with existing testing criteria, it should be noted that all three batches exceeded the batch release specifications for SPC (<2 log CFU/g) outlined by UPSIDE Foods in their FDA consultation dossier. However, it is essential to consider that new standards for cultivated meat should be established, reflecting the unique production processes and microbial risks associated with this novel food category. Traditional meat standards, such as those set for conventional meat production, may not adequately capture the distinct microbial dynamics at play in cultivated meat (Ong et al., 2021), where the closed‐system environment through Stages 1–3, combined with a variable environment during Stage 4, and a more conventional manufacturing environment at Stage 5 can influence both microbial growth and spoilage patterns. Further research is needed to determine the impact of product‐specific characteristics on the microbiological activity, relative risk, and suitable shelf life of cultivated meat products compared to their conventional meat comparators (Ong et al., 2023). This will contribute to the development of a comprehensive risk assessment of the technology, including the importance of cultivated‐meat‐specific variations in water activity, pH, nutrient availability, product composition and microstructure, fat content, background microflora, and the presence of antimicrobial compounds (Dussault et al., 2016; Mejlholm et al., 2010). Microbial guidelines should therefore be established for initial contamination levels based on GMP, accounting for the controlled nature of production, and at the end of the product's shelf life, using spoilage studies that measure both microbial counts and metabolite production, such as volatile organic compounds that indicate spoilage (Casaburi et al., 2015).

The challenge study data included in Figure 5 suggest that the cultivated poultry can satisfy available microbial limits for meat products over a 20‐day storage period, indicating that UPSIDE Foods’ batch release specifications may be conservative. Nevertheless, these limits should be re‐evaluated in light of new standards specifically designed for cultivated meat. Spoilage organisms in meat, such as *Brochothrix thermosphacta* and *Lactobacillus* spp., can significantly affect microbial dynamics, and their metabolites, such as alcohols and esters, should be considered when setting shelf life limits (Ercolini et al., 2011). Further to this, the uncooked Day 0 challenge study samples even met the higher threshold criteria for “low SPC counts” that FSANZ indicates foods should meet if they have received heat treatment processing such as pasteurization or cooking (<3–4 log CFU/g; FSANZ, 2022). This reinforces the need for cultivated meat‐specific guidelines, both for Day 0 and end‐of‐shelf life microbiological safety limits.

A final postproduction consideration is that as cultivated meat products increasingly enter retail settings, consumer behavior will potentially become a critical factor in their microbial contamination risk, as is the case for conventional poultry meat as determined by a quantitative microbiological risk assessment (Khalid et al., 2020). This is a topic made more prominent by GOOD Meat's 3% cultivated shredded chicken becoming available in 2024 in Singapore as the first retail product containing cultivated meat.

## 6 MICROBIAL CONTAMINANTS RELEVANT TO CULTIVATED MEAT

A variety of microorganisms can be present in or introduced into cell cultures used for cultivated meat production. These include bacteria (including mycoplasma), viruses, fungi, and parasites/protozoa. Potential contamination vectors for all these classes of microbes existing in cultivated meat production have been outlined in the previous sections.

In our industry survey, respondents were asked to identify which microbes were the main source of contamination in their cell cultivation over the previous 12 months, with bacteria overwhelmingly cited as the main source of contamination (*n* = 11; Figure 3c). Fungi were identified as a source of contamination to a lesser extent (*n* = 5; Figure 3c), while viral or other contaminants were absent from responses. However, our survey also asked respondents what types of microbes were being tested for during their routine microbial contamination testing practices noted in Figure 2c, which excluded cell line sampling and development. Of 14 respondents to this question, all 14 reported routine bacterial monitoring, five reported routine fungal monitoring, and none reported routine viral monitoring. This reduced routine fungal monitoring and lack of routine viral monitoring may have skewed the identification of primary sources of contamination reported in Figure 3c, as it suggests that testing for fungal, viral, or other contamination outside of Stage 1 was limited. Detection of viral contamination could also be limited as it does not cause food spoilage or impact food product characteristics postharvest (Velebit et al., 2015), which could otherwise prompt testing and detection. Notwithstanding these potential monitoring biases, bacteria are generally recognized as the most common cause of foodborne contamination and illness (Elbehiry et al., 2023), in line with their relative detection frequency in the survey.

### 6.1 Bacteria

As the most common cause of foodborne disease, a range of bacterial contaminants are relevant to cultivated meat production. These present a potential food safety risk that can be introduced through various vectors across all stages of cultivated meat production, as discussed in Section 4 and previously summarized in various publications (FAO & WHO, 2023; Ong & Shatkin, 2024; Sant'Ana et al., 2023).

#### 6.1.1 Bacterial considerations in Stage 1 of production

In Stage 1 of production, bacteria relevant to humans, the environment, and target animal species should be considered for testing due to the necessary exposure of cellular material to the host animal, human staff, and the sourcing and laboratory environments. According to the FAO and WHO (2023), the common pathogenic bacteria that reside in or on animals and their feces that should be considered can include *Listeria*, *E. coli*, *Salmonella*, *Campylobacter*, and *Mycoplasma* species (additional discussion of *Mycoplasma* is included in Section 6.1.4). Additional bacterial species should also be considered where particular target animal species are known reservoirs, for example, *Brucella abortus* for cell sampling from beef cattle (Sant'Ana et al., 2023). Distinct from later stages of production, the use of antibiotics by cultivated meat companies is common to prevent contamination during cell sampling and cell line derivation in Stage 1 (Harsini & Swartz, 2024). Examples of relevant testing protocols for these bacteria can be leveraged from the viral vaccine industry such as those outlined in 9 CFR 113.27(a) (FDA, 1991).

#### 6.1.2 Bacterial considerations in Stages 2 and 3 of production

Direct testing for bacterial contaminants is deemed unnecessary through the Stage 2 and 3 bioprocess as the presence of bacteria can be indirectly detected and addressed based on monitoring for potential indicators of cytopathic effects on the cell substrate as discussed in Section 4.3. An exception to this is *Mycoplasma* species due to their unique growth characteristics, which are discussed in more detail in Section 6.1.4.

#### 6.1.3 Bacterial considerations in Stages 4 and 5 of production

Stages 4 and 5 of production will present similar nonanimal‐based bacterial contamination vectors and risk profiles as conventional meat manufacturing, as end product formulation, processing, and packaging of cultivated meat products are likely to use the same equipment and processes. Therefore, appropriate monitoring and testing requirements can be leveraged from HACCP and GMP for relevant elements of conventional meat processing. Based on a HACCP analysis of cultivated beef burger production, Sant'Ana et al. (2023) identified the most common potential Stage 4 and 5 bacterial contaminants as Shiga‐toxigenic *E. coli*, *Salmonella*, *L. monocytogenes*, and *S. aureus*.

#### 6.1.4 *Mycoplasma* contamination of cultivated meat production

Mycoplasma are a genus of bacteria characterized by their lack of a rigid cell wall and are considered to be the smallest self‐replicating organisms known currently, characteristics that make them a major contamination hazard for cultivated meat production. Mycoplasma are difficult to eradicate during biomanufacturing due to their ability to pass through commonly used 0.45‐µm filters and their resistance to most antibiotics commonly used in cell culture (Drexler & Uphoff, 2002; Nikfarjam & Farzaneh, 2012). For mycoplasma to present a direct food safety hazard, a live microorganism would have to persist in the final cultivated meat product and be capable of infecting a consumer via the oral route; however, there are no reported cases in the clinical literature of mycoplasma infection via the oral route in humans (FAO, 2022). An alternate risk, though one with only select known examples, is that mycoplasma contamination can cause genomic, genetic, and phenotypic instability in cell lines (He et al., 2023; Ji et al., 2019), which could lead to the expression of novel toxins in cultivated meat cell lines that persist in the final product (FAO, 2022). The general hazard of genetic instability‐induced toxin production is not unique to cultivated meat production, and best practice management strategies can be referred to from cellular therapeutics and biosimilar industries (FAO, 2022).

Tests to detect the presence of mycoplasma are commercially available and recommended under the GCCP framework (Bal‐Price & Coecke, 2011; Pamies et al., 2022). The industry survey questions did not specifically request information on *Mycoplasma* as a distinct microbial test option separate from other bacterial testing, and as a result, only six survey respondents proactively indicated they conduct *Mycoplasma* testing where additional information on specific tests was requested. Despite this incomplete dataset, the survey did indicate that variation in testing protocols is a consideration for the sector as one respondent indicated that they conduct *Mycoplasma* testing on all master cell bank samples and working cell bank samples, which is in line with GCCP, while another only tested cell cultures displaying visual indications of *Mycoplasma* contamination, which is not in line with GCCP (Pamies et al., 2022). Again, precedent from the viral vaccine manufacture sector can be used to inform appropriate testing methods for cultivated meat production such as PCR‐based testing assays (Duguid et al., 2009; Lawrence et al., 2010). These PCR assays are highly specific and sensitive and can be used for frequent testing, which may be necessary due to high rates of mycoplasma contamination in cell culture across both research and manufacturing (Nikfarjam & Farzaneh, 2012). Of note, the six most prevalent strains of mycoplasma in cell culture contaminations are *Mycoplasma arginini*, *Mycoplasma fermentans*, *Mycoplasma hominis*, *Mycoplasma hyorhinis*, *Mycoplasma orale*, and *Acholeplasma laidlawii* (Geraghty et al., 2014), which have variable pathogenicity in humans (Afshar et al., 2008; Taylor‐Robinson, 1996; Taylor‐Robinson & Bébéar, 1997). Additional strains may be relevant for manufacturers and regulators to consider based on analysis of their risk potential.

### 6.2 Fungi

Fungal pathogens such as yeasts and molds are a food safety threat due to secondary metabolites produced by some species, called mycotoxins, which have high toxicity and can be absorbed through the digestive tract when contaminated products are consumed (Kępińska‐Pacelik & Biel, 2021). There are three pathways by which conventional meat products can be contaminated with mycotoxins and present a food safety risk; they can accumulate in the meat of farm animals that consume contaminated feed, can be introduced via contaminated spices or other raw materials added to processed meat products, and have been demonstrated to be directly produced by molds on the surface of dry‐cured meat products (Pleadin et al., 2021).

Mycotoxins could theoretically be introduced to cultivated meat from the vector of fungal‐contaminated culture media components akin to the vector of contaminated animal feed. However, this vector should be monitored and controlled by media suppliers and cultivated meat companies through existing Good Agricultural Practices (GAP), GMP, and HACCP monitoring systems. If mycotoxins were able to reach the production environment, their presence would likely be deleterious to cellular viability (Smith et al., 2016), which would facilitate their detection during Stages 1–3 of production. The direct growth of molds on the meat surface and the subsequent production of mycotoxins generally occur during dry‐curing of meat, which is characterized by constantly high relative air humidity and moderate to high temperatures (Pleadin et al., 2021). This vector would be relevant to cultivated meat products that undergo similar dry‐curing processes where mold growth is facilitated. Mold growth dynamics and relevant food safety practices during dry‐curing of conventional and cultivated meat could be impacted by differences in product composition and would need to be characterized, as discussed in Section 5. Finally, the addition of further ingredients to cultivated meat products, such as spices, would present the same mycotoxin vector as for conventional meat products at Stage 5 of production and be controlled by the same food safety practices.

Similar to bacteria, fungi are relevant to consider for testing at Stage 1 when deriving or working with new cell lines that will be banked due to potential contamination, and relevant testing protocol examples can be leveraged from the viral vaccine industry.

Additionally, fungal contamination can occur via the production environment in Stages 2–5. For example, a study by Martin et al. (2012) identified the presence of fungal spores of *Aspergillus*, *Penicillium*, and *Cladosporium* genera in the air and surfaces of a pharmaceutical stem cell production facility, originating from personnel manipulation and air microbiota, which would be instructive for relevant fungal microbes that could be present in a cultivated meat facility. These genera are also among the most common fungal contaminants of conventional meats and meat products and can lead to product spoilage or present a food safety risk to consumers via mycotoxin production (Pleadin et al., 2021).

### 6.3 Viruses

A variety of viruses can potentially be present in cells obtained in Stage 1 of production or introduced from personnel, equipment, the environment, or other process inputs through all stages of production. Potential virus sources as outlined in Barone et al. (2020) include endogenous retroviruses; other adventitious viruses known to exist in the species of origin (species‐specific viruses); viruses acquired from personnel (e.g., respiratory viruses) during routine cell culture; viruses acquired from cross‐contamination from other cell lines in the laboratory or facility; and viruses in culture media ingredients that are animal sourced or nonanimal sourced.

#### 6.3.1 Viral considerations in Stage 1 of production

In Stage 1 of production, testing to detect viruses that can infect or otherwise be harmful to consumers is an important consideration. There are a few key points to consider for understanding the viral infection risk to consumers. First, the species barrier between humans and an animal viral pathogen will reduce the risk of zoonotic transmission; however, it is estimated that 60%–75% of human infectious diseases are originally circulated in nonhuman animal species, while transmission of human‐specific viruses to animal cell lines is also possible (Ellwanger & Chies, 2021). Both vectors need to be considered by manufacturers and regulators as highlighted in the publicly available FDA safety dossiers prepared by UPSIDE Foods (Schulze, 2021).

In general, viral testing employing solely molecular biology and in vitro methods rather than testing in vivo has been deemed sufficient for related industries like vaccine manufacturing (Gombold et al., 2014). In the event of a positive test, a crucial consideration is whether the detected virus could be infectious to humans. Relevant testing pipelines have previously been developed in the vaccine industry that could be adapted to cultivated meat production purposes (FDA, 2010; Gombold et al., 2014). Removal or inactivation of endogenous retroviruses can also be achieved, using genomic modification techniques as described by Yang et al. (2015).

Some animal species may be known potential hosts for well‐characterized viruses (e.g., bovine viral diarrhea virus, bovine leukemia virus [BLV]) that could contaminate cultivated meat production if they are transferred into a cell line from an infected animal through the vector of initial cell sourcing in Stage 1 of production. Regulations exist in some jurisdictions outlining relevant viruses and testing methods to consider for some of the most commonly consumed species, such as 9 CFR 113.47 (FDA, 1995). As additional species, including exotic animals, are explored for cultivated meat purposes, relevant hazardous viruses will need to be considered.

It is currently unclear how many animal viral pathogens are currently associated with human disease or may become associated with disease based on future research. Development of databases that catalog animal viruses that are associated with human disease by manufacturers or regulators could assist manufacturers and cell line developers in conducting case‐by‐case risk assessments dependent on the virus and species. In these instances, a risk assessment may also take into consideration the known ability of a virus to propagate in vitro as, for instance, hepatitis E virus may be transmissible to humans via pork liver via consumption (Pavio et al., 2014) but has been notably difficult to propagate in vitro (Meister et al., 2019). Another scenario may include testing for hepatitis E or A virus when sourcing cells from live bivalve shellfish. An opportunity also exists for the industry to create, validate, and adopt additional antibody‐ and/or PCR‐based assays for species‐specific viral testing of viruses deemed a potential hazard.

There is accumulating evidence that some viruses traditionally labeled as species specific (e.g., BLV) can be zoonotic via foodborne transmission and potentially human pathogenic (Buehring et al., 2019, 2015). BLV is common in many cattle populations around the world. In the conventional meat industry, only infected animals that develop leukemia or lymphoma are excluded from the food system. As discussed earlier, cells that are sourced from healthy animals that have not been excluded from the food supply chain, such as those recommended for viral vaccine manufacturers (FDA, 2010), would reduce the risk of BLV being present in cells obtained for cultivated meat purposes. However, it is unclear if in vitro culture of cells from non‐excluded cattle could propagate BLV in vitro and whether that virus would be human pathogenic. Further research is needed to understand this possibility (Ong et al., 2021).

Species‐specific viruses are also a consideration when animal serum is used in a culture medium as demonstrated by GOOD Meat's use of fetal bovine serum (FBS) and subsequent testing for bovine viruses (Licari, 2022) informed by the US Department of Agriculture, 9 CFR 113.53(C) (US Department of Agriculture, 2024).

Another unique potential vector of viral contamination is a viral construct‐mediated genetic modification to improve a cell line's characteristics. Most commonly, this technique is used to extend the proliferative capacity of cells, referred to as cell line “immortalization.” Some viral constructs, such as lentiviruses or retroviruses, could represent a contamination vector if they unintentionally undergo recombination to form a replication‐competent and pathogenic virus during integration into the cell line and are subsequently undetected through the production process. Newer generations of viral constructs remove genes from the construct that contribute to virulence in the native virus, separate the genes required for replication into different plasmids that are not all integrated into the host cell DNA, and can include self‐inactivating sequences to reduce the viral vectors expression after integration into the host DNA (Schlimgen et al., 2016). Together, these modifications substantially reduce but do not eliminate the risk of replication‐competent viruses being introduced (Alteri et al., 2016); however, it must be noted that subsequent cultivated meat production monitoring should identify such an event.

Notwithstanding the above considerations of the low‐probability nature of this risk, a 2023 cultivated meat industry survey on cell line use highlighted that only 29% (*n* = 10/34) of respondents would employ permanent genome alteration for commercial cell line development (Ravikumar & Powell, 2023). The specific method of genome alteration companies would employ was not noted in the survey of Ravikumar and Powell (2023), but the use of viral constructs is only one of many chemical, physical, or biological alteration techniques. In practice, the potential risk of viral construct‐based genome modification was ruled out as an acceptable hazard to employ in UPSIDE Foods production process in their FDA cell culture consultation dossier (Schulze, 2021), suggesting that companies may choose to avoid exposure to this vector in their processes.

#### 6.3.2 Viral considerations in Stages 2 and 3 of production

If viruses are undetected in cell lines at Stage 1 of production or subsequently introduced through other vectors, viruses can replicate in cells during Stages 2 and 3 of production, resulting in impacted cellular growth and the potential for viral particles to persist in final cultivated meat products. However, similar to consideration of bacterial testing, direct testing for viral contaminants is deemed unnecessary through the Stage 2 and 3 bioprocess as the presence of viruses can be indirectly detected and addressed based on monitoring for potential indicators of cytopathic effects on the cell substrate as discussed in Section 4.3.

#### 6.3.3 Viral considerations in Stages 4 and 5 of production

At Stage 4 of production, there is a loss of cell viability during the harvesting of cells, which removes the potential for viral replication as they cannot replicate in a dead host. However, chilled and frozen storage of foods can preserve inactive virions for months or years; therefore, a level of risk persists similar to conventional meat processing (Bosch et al., 2018). Stages 4 and 5 of production will present the most similar viral contamination vectors and risk profiles as conventional meat manufacturing, as end product formulation, processing, and packaging of cultivated meat products are likely to use the same equipment and processes. Therefore, appropriate monitoring and testing requirements can be leveraged from HACCP and GMP for relevant elements of conventional meat processing. The two most common foodborne viruses are norovirus and hepatitis A virus, with epidemiological data related to raw meat demonstrating that infected handlers at slaughterhouses are repeatedly involved in the transmission of these viruses if they practice poor personal hygiene (Todd et al., 2007a, 2007b). In food processing, viruses are effectively destroyed by temperatures above 80°C (Zhang et al., 2019). Exceptions may include instances where cultivated meat products could be harvested and immediately served raw (e.g., steak tartare, sushi) prior to a complete loss of cell viability or shortly after a loss of cell viability. Cultivated meat will also travel through the acidic gastrointestinal tract, which would reduce the opportunity for transmission of many viruses (Li et al., 2019; Lieleg et al., 2012; Piazza, 1964; Scholtissek, 1985), though some like noroviruses that infect the intestines have developed mechanisms to circumvent this (Tenge et al., 2021). Therefore, maintenance of cooking and hygiene practices associated with conventional meat would also reduce the incidence of viral contamination reaching consumers should control measures at Stage 5 be ineffective.

### 6.4 Prions

A prion is a type of protein that can cause disease in animals and humans by triggering normally healthy proteins in the brain to fold abnormally. The prion mode of action is very different from bacteria and viruses as they are simply proteins, devoid of any genetic material.

Prions are the causative agent behind transmissible spongiform encephalopathies (TSEs). Forms of TSEs have occurred in cows, sheep, goats, lemurs, elk, deer, cats, and mink. TSEs are rare but are fatal and untreatable. The most documented form of TSE in humans is variant Creutzfeldt–Jakob Disease (vCJD), which is acquired from eating tissues from cows with bovine spongiform encephalopathy (BSE). In sheep, TSE manifests as scrapie, which has little evidence of transmissibility to humans. Typically, TSEs are most detectable in tissues of the central nervous system (CNS), which contain cells that propagate prions more readily than other tissues, but they can also be found throughout other bodily tissues or fluids. It is unlikely that cultivated meat products would originate from CNS and other TSE‐containing tissues, but the risk of biological samples obtained by cultivated meat manufacturers originating from or becoming in contact with TSE‐containing tissues is not zero. Thus, TSEs could pose a risk if they were present in the cell lines used in cultivated meat manufacturing, as TSEs are known to spread through consumption.

Another potential exposure risk could arise if BSE were transmissible through the use of FBS as a cell culture media supplement. A variety of evidence suggests BSE is not transmissible via bovine serum (WHO, 2006; Wrathall et al., 2002). It is unclear whether sera from other TSE‐positive animals may contain prions or whether those prions may be transmissible in vitro.

There is no deemed risk of prions originating from animal populations with no documented history of TSEs, meaning biological samples used for cell line derivation, components of media, or processing aids can be obtained from healthy animals or animal populations with no known history of TSEs or symptoms of TSE disorders, which typically involve gait abnormalities and weight loss. The World Organisation for Animal Health maintains the official BSE status of countries or zones for member countries (World Organisation for Animal Health, 2023).

If cells or animal serum such as FBS are used and the origins or quality assurance of the specimen cannot be validated, as suggested for GCCP (Bal‐Price & Coecke, 2011), then tests are available for prior detection, such as real‐time quaking‐induced conversion and protein misfolding cyclic amplification.

### 6.5 Parasites/protozoa

Protozoa and parasite contamination in animal cell cultures have been cited, but their frequency is exceedingly rare (Fogh et al., 1971) and they pose an overall low risk in Stage 1 and throughout all subsequent process stages. Routine process monitoring for parasites and protozoa is similar to that described for bacteria and fungi above. In their theoretical HACCP analysis of cultivated burger products, Sant'Ana et al. (2023) highlighted the protozoa *Toxoplasma gondii* and parasite *Cryptosporidium parvum* as biological hazards that could be introduced through the vector of the donor animal supplying initial cells to the process.

## 7 CONCLUSIONS AND FUTURE PERSPECTIVES

Based on this review study, several trends in cultivated meat production are apparent. Personnel, equipment, and the environment were identified as the primary vectors of microbial contamination; bacteria were the most reported and most consistently monitored contaminant; and contamination rates are variable and higher than might be expected based on the theoretical potential of cultivated meats. This expectation of low contamination is due to the potential of cultivated meats for an aseptic bioprocess in Stages 1–3 and the lack of animal and fecal vectors of contamination during Stages 4 and 5 of production as compared to conventional meat production. The higher microbial contamination reported in our survey is almost certainly due to the industry's nascent status and rapid scale‐up, with many companies still establishing and embedding relevant food safety management regimes and staff training. These are not novel challenges, and to manage these contamination risks, the sector can borrow and adapt GMP and HACCP knowledge and practices from conventional meat production to reduce contamination incidence during Stages 4 and 5 of production while similarly borrowing and adapting GCCP, GMP, and/or HACCP knowledge and practices from the biopharmaceutical industry during Stages 1–4 of production. Conversely, it may become apparent that additional or novel technologies and practices to maintain sterile cell culture conditions could be more cost‐effective compared to following biopharmaceutical industry practices such as the use of classified clean rooms with HEPA‐filtered air for some or all stages of production, and research to this end is warranted.

Considering cultivated meat end products, the current lack of public data on their microbial growth profiles makes it difficult to develop comprehensive data‐driven industry‐level microbiological standards as highlighted by the limited conclusions that could be drawn regarding the *Listeria* challenge data reported herein. To develop such standards, it is necessary to determine the growth potential of spoilage and pathogenic microorganisms in different cultivated meat products, taking into account factors such as product composition and microstructure, pH, water activity, fat content, and background microflora. This requires a collaborative effort within the industry, including the formation of research consortia and partnerships with academic institutions, to facilitate data sharing and conduct exhaustive food safety studies that can be used to develop standardized test methods and data‐driven safety guidelines. These studies should explore the effectiveness of different preservation techniques and storage conditions in controlling microbial growth to maintain product quality. In addition, microbiological prediction tools and predictive modeling can be of great value in informing the development of risk‐based microbiological standards by simulating microbial behavior under different scenarios.

It is important to recognize that cultivated meat's distinct production methods and potential microbial risks will warrant distinct microbiological standards from conventional meat production. Therefore, until sufficient data are available to set appropriate company‐agnostic cultivated meat‐specific standards, regulatory authorities should be responsive to new safety data becoming available and new bioprocess innovations being integrated into commercial production.

Finally, a downstream food safety consideration is the behavior of consumers toward handling, storing, and cooking cultivated meat compared to conventional meat and how that aligns with actual microbial risk and labeling requirements, which will become increasingly important as more cultivated meat products become available through retail channels.

## AUTHOR CONTRIBUTIONS

**Dean Powell**: Conceptualization; methodology; writing—review and editing; writing—original draft; funding acquisition; project administration; investigation; formal analysis; data curation; visualization. **Dan Li**: Visualization; writing—review and editing; formal analysis; data curation; conceptualization; investigation. **Ben Smith**: Conceptualization; writing—review and editing; funding acquisition; methodology; investigation; resources. **Wei Ning Chen**: Funding acquisition; writing—review and editing.

## CONFLICT OF INTEREST STATEMENT

The authors declare no conflicts of interest.
